# Changes in T-cell subsets and their association with disease severity in patients with alopecia areata

**DOI:** 10.55730/1300-0144.6348

**Published:** 2026-04-12

**Authors:** Hatice TÜRK DAĞI, Hülya ÖZDEMİR, Mehmet AKYÜREK, Fatma TUNÇEZ AKYÜREK, Hasibe ARTAÇ

**Affiliations:** 1 Department of Medical Microbiology, Faculty of Medicine, Selçuk University, Konya, Turkiye; 2 Department of Medical Biology, Faculty of Medicine, Selçuk University, Konya, Turkiye; 3 Department of Dermatology, Osmaniye State Hospital, Osmaniye, Turkiye; 4 Department of Dermatology, Faculty of Medicine, Selçuk University, Konya, Turkiye; 5 Division of Immunology and Allergy, Department of Pediatrics, Faculty of Medicine, Selçuk University, Konya, Turkiye

**Keywords:** Alopecia areata, follicular T-cell populations, regulatory T-cell populations, T-cell subsets

## Abstract

**Background/aim:**

Alopecia areata (AA) is a nonscarring hair loss disorder characterized by genetic susceptibility, environmental triggers, and T-cell–mediated autoimmunity. While various CD4^+^ and CD8^+^ T-cell subsets are implicated in its pathogenesis, the roles of follicular T-cell (Tfh), peripheral T-cell (Tph), and regulatory T-cell (Treg) subsets–particularly within the CD8^+^ population–remain incompletely defined. This study aimed to characterize circulating CD4^+^ and CD8^+^ T-cell subsets in Turkish patients with AA and to evaluate their associations with disease activity, duration, and severity.

**Materials and methods:**

A total of 40 patients with AA and 40 healthy controls were enrolled. CD4^+^ and CD8^+^ T-cell subsets—including Th1, Th2, Th17, Th22, Tc1, Tc2, Tc17, Tc22, Treg, Tfh, and Tph cells—were analyzed by flow cytometry based on surface marker expression and intracellular cytokine profiles. Disease severity was assessed using the Severity of Alopecia Tool (SALT) score, and disease activity was evaluated by the hair pull test.

**Results:**

Patients with AA exhibited significantly lower frequencies of Tc17 and Tc22 cells than healthy controls. CD4^+^ Tfh cell levels were positively correlated with disease duration. CD4^+^ Tregs, resting Tregs, CD3^+^IL-4^+^ cells, and Tc2 cells were positively associated with SALT scores, whereas CD4^+^ and CD8^+^ Tph cells were inversely associated. CD8^+^ T-cell populations, CD4^+^ Treg subsets, CD3^+^IFN-γ^+^ cells, Th1 cells, and Th2 cells were associated with disease activity, as assessed by the hair pull test.

**Conclusion:**

Alopecia areata is associated with alterations in circulating T-cell subsets, particularly Tc17 and Tc22 subsets. Selected cytokine-producing and regulatory T-cell subsets are associated with disease activity. These findings support a heterogeneous and dynamic immune profile in AA and highlight the importance of multivariate approaches in identifying independent immunological correlates. Further studies integrating tissue-level analysis and longitudinal follow-up are needed to clarify the underlying mechanisms.

## 1. Introduction

Alopecia areata (AA) is a nonscarring hair loss disorder with a relatively high prevalence across all ages, sexes, and ethnic backgrounds. It affects approximately 2% of the population and occurs equally in both sexes [1,2].

Clinically, AA is typically characterized by well-demarcated patches of hair loss on the scalp without inflammation or scarring. Patients with active disease often have a positive hair pull test, particularly around the lesion. In severe cases, the disease may involve the entire scalp (alopecia totalis [AT]) or all body hair (alopecia universalis [AU]). In some patients, the pattern may be band-like, as observed in ophiasis, or diffuse. Nail abnormalities are observed in 10%–15% of patients and may reach 44% in certain populations [3].

The pathogenesis of alopecia areata involves genetic predisposition, environmental factors, and autoimmune processes. Disruption of the hair follicle immune privilege plays a pivotal role, resulting in predominant T-cell infiltration [4–6]. In the pathogenesis of AA, CD8^+^ NKG2D^+^ T-cell populations play a pivotal role, along with a spectrum of inflammatory cells, including regulatory T-cell (Treg), dendritic cells, natural killer T-cell populations, tissue-resident memory T-cell populations, and mast cells. Activation of CD8^+^ NKG2D^+^ T-cell populations induces IFN-γ production via JAK1/3 signaling, which, in turn, stimulates the release of IL-15 from follicular epithelial cells through JAK1/2 signaling. IL-15 further enhances IFN-γ secretion, creating a self-amplifying loop that sustains inflammation. In parallel, IL-12/23–mediated JAK-STAT activation promotes the release of Th1- and Th17-related cytokines (IFN-γ, IL-17A, and IL-22), thereby amplifying the follicular immune response [7]. Various studies have demonstrated the involvement of Th1, Th2, Th17, and Th22 subsets, as well as CD4^+^ regulatory T (Treg) cells and cytotoxic T (Tc) cells. However, the roles of CD8^+^ Treg cells, follicular T (Tfh) cells, and peripheral T (Tph) cells have not been thoroughly investigated. Moreover, immune mechanisms and immune cell profiles in Turkish patients with AA have not been specifically investigated. The inclusion of a Turkish cohort will offer a unique perspective, as regional genetic polymorphisms and environmental exposures may influence immune phenotypes in AA patients. Therefore, this study aimed to characterize T-cell subsets in Turkish patients with AA and to explore their clinical correlations.

## 2. Materials and methods

### 2.1. Sample size

To detect a statistically significant difference in T-cell subsets between patients with AA and healthy controls, a two-tailed hypothesis test using an independent samples t-test was planned at a significance level of 5%, with 80% statistical power and an effect size of 0.7 (Cohen’s d = 0.7). Accordingly, the minimum sample size was calculated as 68 individuals in total, with at least 34 individuals in each group. Considering a possible 10% data loss, 40 patients with AA and 40 healthy individuals were included in the study.

The exclusion criteria included age under 16 years, the presence of autoimmune comorbidities, the use of immunosuppressive drugs, systemic treatment for AA (systemic corticosteroids, methotrexate, cyclosporine, or JAK inhibitors), ongoing infection, pregnancy, or lactation.

Demographic and clinical data, including age, sex, disease duration and severity, previous treatments, and contact information, were collected from all participants. Disease severity was evaluated using the Severity of Alopecia Tool (SALT) score, which is calculated based on hair loss percentages in four regions of the scalp: vertex (40%), right (18%), left (18%), and posterior (24%). Nail involvement was also recorded.

The hair pull test was performed by a single experienced dermatologist using a standardized clinical procedure. Patients were instructed to avoid shampooing for 3 days prior to the test. The test was performed by gently pulling approximately 50–60 strands of hair from the border of the alopecia area. A positive result was defined as the separation of more than 10% of the pulled hairs [8].

### 2.2. Flow cytometry analysis

CD4^+^ T-cell subsets (Th1, Th2, Th17, Th22, CD4^+^ Treg, CD4^+^ Tfh, and CD4^+^ Tph) and CD8^+^ T-cell subsets (Tc1, Tc2, Tc17, Tc22, CD8^+^ Treg, CD8^+^ Tfh, and CD8^+^ Tph) were identified using fluorescently labeled monoclonal antibodies based on surface marker expression and/or intracellular cytokine levels (Supplementary Table 1). Details of all antibody clones, fluorochrome conjugates, and manufacturers are provided in Supplementary Table 2.

Peripheral blood mononuclear cells (PBMCs) were isolated by density gradient centrifugation using Lymphopure (BioLegend, San Diego, CA, USA) and analyzed immediately after isolation. Cell viability and concentration were determined using the trypan blue exclusion method with a Thoma hemocytometer.

Cells were adjusted to a concentration of 1 × 10^6^ cells/mL, and a cell activation cocktail containing brefeldin A (BioLegend, San Diego, CA, USA) was added at 2 μL per 1 × 10^6^ cells. Cells were incubated for 6 h at 37 °C in a humidified 5% CO_2_ incubator. Surface staining was performed using a combined CD3/CD4/CD8 antibody panel for 20 min at room temperature in the dark. Subsequently, cells were fixed and permeabilized using Cyto-Fast Fix/Perm solution (BioLegend, San Diego, CA, USA) according to the manufacturer’s instructions. Cells were subsequently washed twice with 1X Cyto-Fast Perm wash solution (BioLegend, San Diego, CA, USA). Intracellular cytokine staining was performed using antibodies against IL-4, IL-17A, IFN-γ, and IL-22.

For Treg analysis, PBMCs were stained extracellularly with CD3, CD4, CD8, CD25, and CD45RA antibodies, fixed with Foxp3 fixation buffer, permeabilized (BD Biosciences, Franklin Lakes, NJ, USA), and stained intracellularly with an anti-Foxp3 antibody. For Tfh and Tph analyses, cells were stained with CD3, CD4, CD8, CXCR5, and PD-1 antibodies.

Data acquisition was performed using a 10-color BD FACS Aria III flow cytometer (BD Biosciences, Franklin Lakes, NJ, USA), and the data were analyzed using FACS Diva software (version 6.1.3; BD Biosciences, Franklin Lakes, NJ, USA). The gating strategy for identifying T-cell subsets is illustrated in Supplementary Figures 1–3.

### 2.3. Statistical analysis

All statistical analyses were conducted using R version 3.6.0 (The R Foundation for Statistical Computing, Vienna, Austria; https://www.r-project.org). Data normality was assessed using the Shapiro-Wilk test and visualized using Q-Q plots. The homogeneity of variance was tested using Levene’s test. Outliers were identified using Dixon’s Q test and excluded if necessary.

Descriptive statistics for numerical variables were presented as the mean ± standard deviation (SD), median (range: minimum–maximum), or median (interquartile range [IQR], 1st–3rd quartile), whereas categorical variables were expressed as the frequency (n) and percentage (%).

Comparisons between patients with AA and healthy controls were performed using independent samples t tests for normally distributed variables and the Mann-Whitney U test for nonnormally distributed variables. Welch’s t test was used in cases of unequal variance.

Correlations between T-cell subgroups and hematological parameters were assessed using Pearson or Spearman’s rho correlation coefficients, depending on the data distribution. Associations between disease duration, SALT scores, and T-cell subsets were also evaluated.

Multivariable analyses were performed to assess independent associations between T-cell subsets and clinical outcomes. For SALT score, generalized linear models (GLMs) with a gamma distribution and a log link function were used due to the nonnormal distribution of the outcome variable. For pull test status, multiple logistic regression models were applied. All models were adjusted for age, sex, and disease duration, and additionally adjusted for SALT score in logistic regression models.

A p-value of <0.05 was considered statistically significant. Statistically significant findings were visualized using boxplots and scatter plots.

Ethical approval was obtained from the Local Ethics Committee (decision no: 2021/111, dated 24 February 2021). Informed consent was obtained from all participants included in the study.

## 3. Results

A total of 40 patients with AA (13 females, 27 males; mean age: 30.75 ± 11.97 years) and 40 healthy controls (16 females, 24 males; mean age: 29.85 ± 11.06 years) were included in the study. The age and sex distributions between the two groups were similar (p = 0.728 and p = 0.642, respectively) (Table 1).

**Figure 1:**
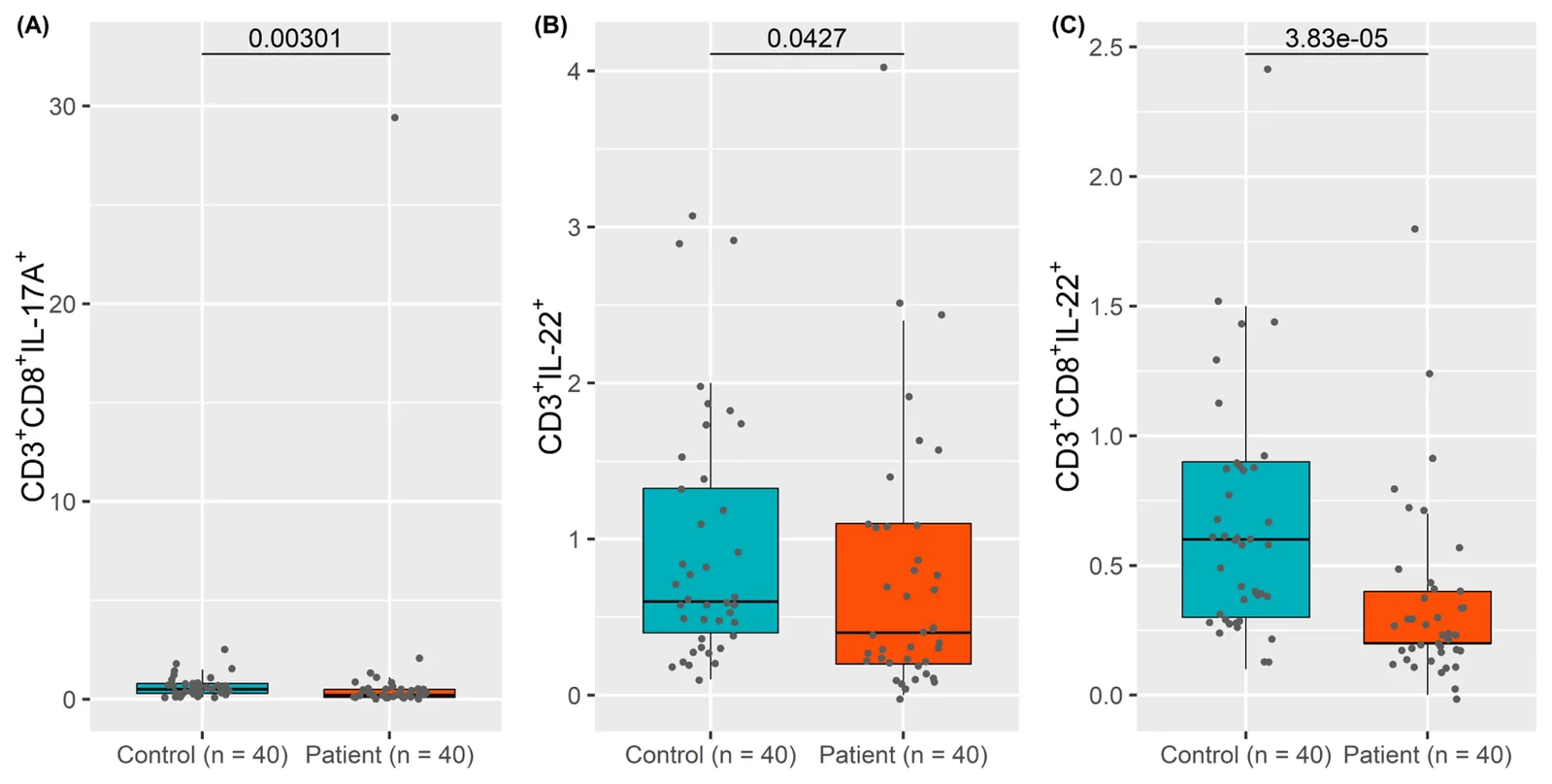
Box plots showing (A) CD3^+^ CD8^+^ IL-17A^+^ levels (p = 0.003), (B) CD3^+^ IL-22^+^ levels (p = 0.043), and (C) CD3^+^ CD8^+^ IL-22^+^ levels (p < 0.001) in patients and controls.

There was no statistically significant difference between the patients and controls regarding total T cells, CD4^+^ T cells, Th1 cells, Th2 cells, Th17 cells, Th22 cells, CD4^+^ Treg cells, CD4^+^ Tfh cells, CD4^+^ Tph cells, CD8^+^ T cells, Tc1 cells, Tc2 cells, CD8^+^ Treg cells, CD8^+^ Tfh cells, or CD8^+^ Tph cells (Table 2). However, significantly lower percentages of Tc17 (p = 0.003), total IL-22^+^ T cells (p = 0.043), and Tc22 (p < 0.001) were observed in patients with AA than in controls (Figures 1A–1C). After adjustment for multiple comparisons using the Benjamini–Hochberg method, the differences in Tc17 (p = 0.003) and Tc22 (p = 0.012) frequencies remained statistically significant.

**Figure 2:**
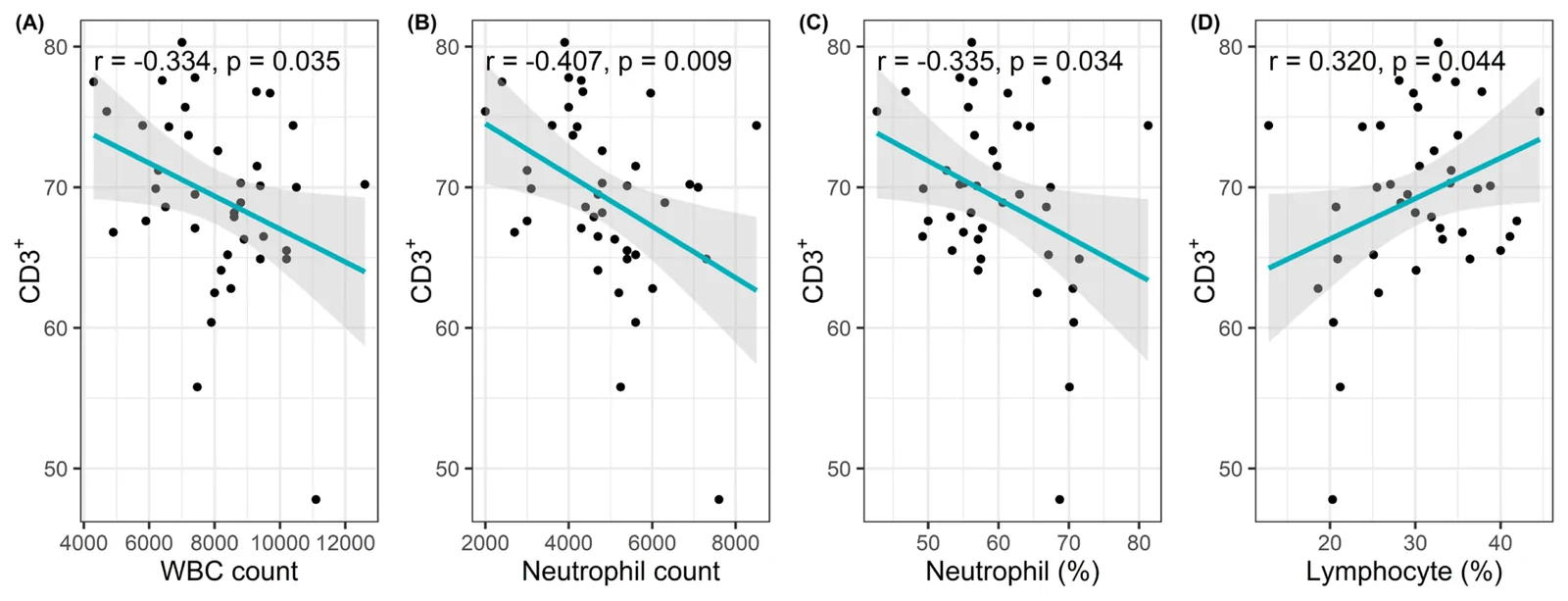
Scatter plots showing the relationships between total CD3^+^ T-cell percentage and (A) WBC count, (B) neutrophil count, (C) neutrophil percentage, and (D) lymphocyte percentage. The r value represents Pearson’s correlation coefficient. The blue line represents the linear regression line, and the gray area indicates the confidence interval.

Significant negative correlations were detected between total CD3^+^ T cells and white blood cell (WBC) count (r = −0.334; p = 0.035), neutrophil count (r = −0.341; p = 0.009), and neutrophil percentage (r = −0.335; p = 0.034), whereas a positive correlation was detected with lymphocyte percentage (r = 0.32; p = 0.044) (Figures 2A–2D).

**Figure 3:**
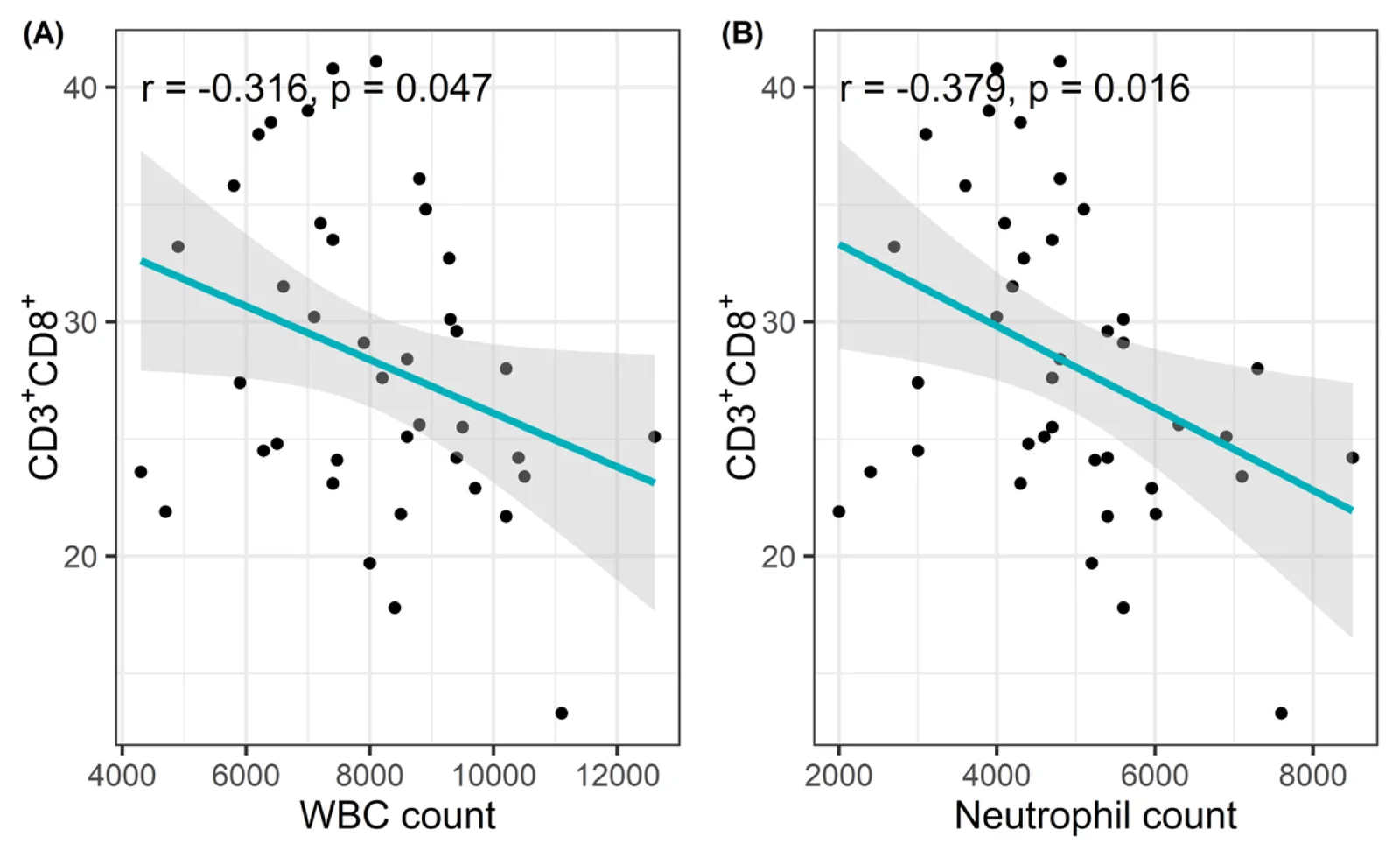
Scatter plots showing the relationships between total cytotoxic T cells and (A) leukocyte count and (B) neutrophil count. The r value represents Pearson’s correlation coefficient. The blue line represents the linear regression line, and the gray area indicates the confidence interval.

The total number of Tc cells was negatively correlated with WBC count (r = −0.316; p = 0.047) and neutrophil count (r = −0.379; p = 0.016) (Figures 3A and 3B).

**Figure 4:**
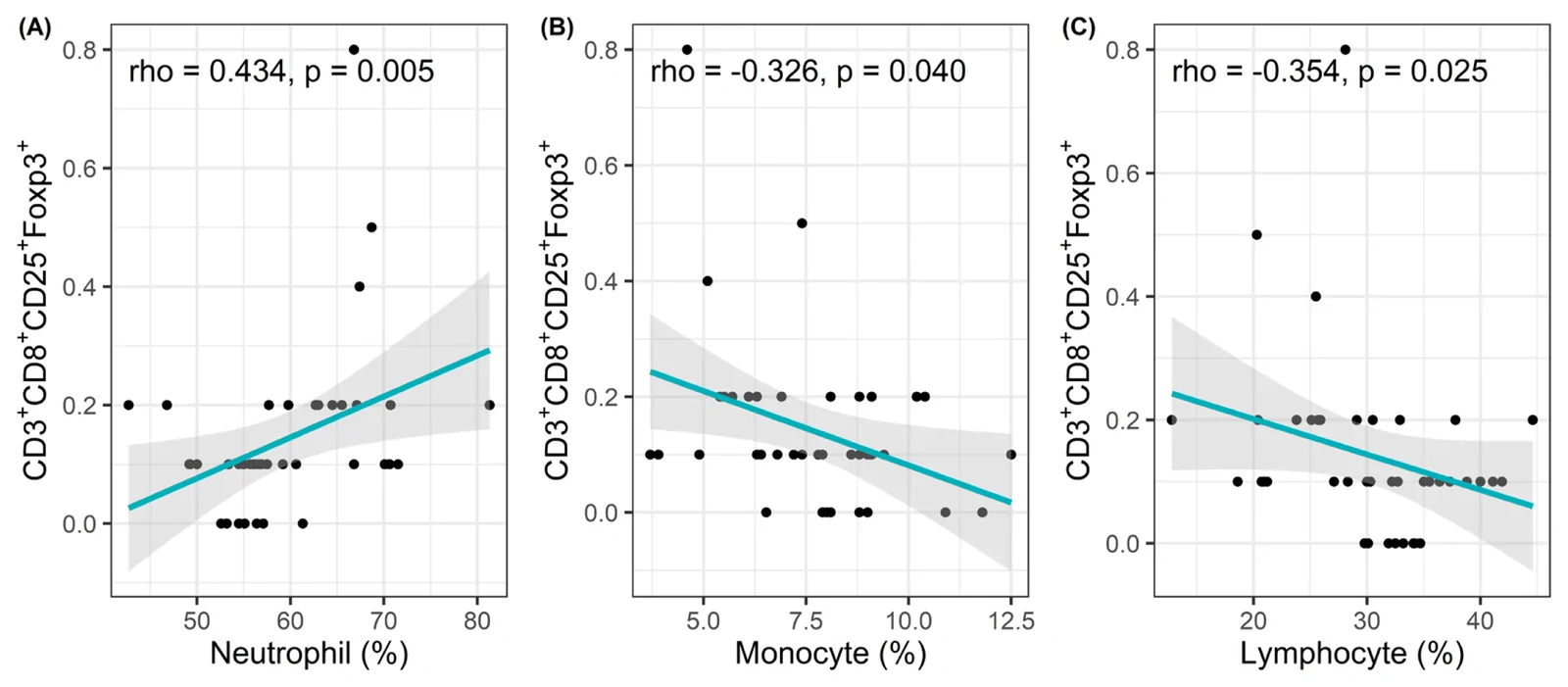
Scatter plots showing the correlations between CD8^+^ Treg cells and (A) neutrophil percentage, (B) monocyte percentage, and (C) lymphocyte percentage. The *ρ* value represents Spearman’s rho correlation coefficient. The blue line represents the linear regression line, and the gray area represents the confidence interval.

The percentage of CD8^+^ Treg cells was significantly positively correlated with neutrophil percentage (Spearman’s rho = 0.434; p = 0.005) and significantly negatively correlated with monocyte percentage (Spearman’s rho = −0.326; p = 0.040) and lymphocyte percentage (Spearman’s rho = −0.354; p = 0.020) (Figures 4A–4C).

**Figure 5:**
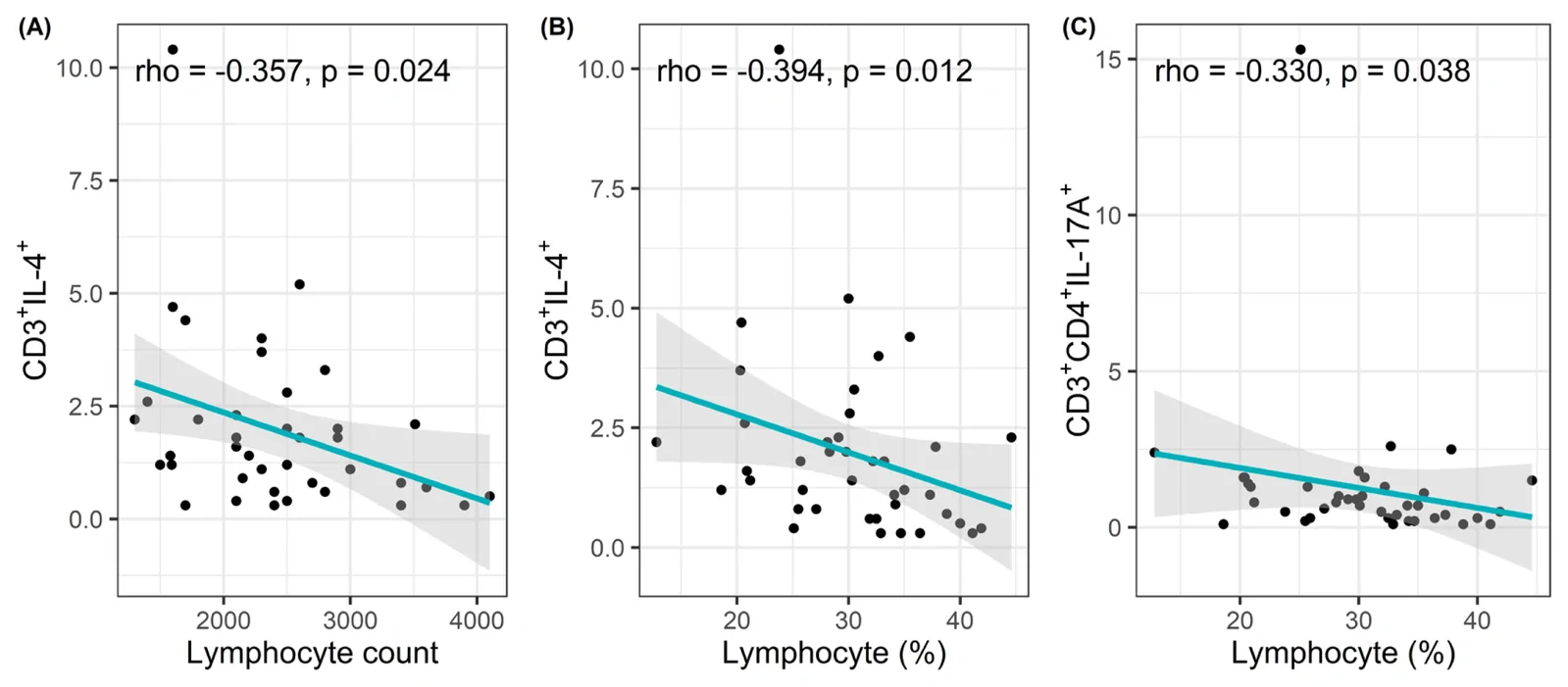
Scatter plots showing correlations between total IL-4^+^ T cells and (A) lymphocyte count, (B) lymphocyte percentage, and (C) Th17 cell percentage and lymphocyte percentage. The *ρ* value represents Spearman’s rho correlation coefficient. The blue line represents the linear regression line, and the gray area represents the confidence interval.

A significant negative correlation was found between IL-4^+^ total T-cell count and lymphocyte count (Spearman’s rho = −0.357; p = 0.024) and lymphocyte percentage (Spearman’s rho = −0.394; p = 0.012), as well as between Th17 percentage and lymphocyte percentage (Spearman’s rho = −0.33; p = 0.038) (Figures 5A–5C). No significant associations were detected between other T-cell subpopulations and hematological parameters in patients with AA (all p > 0.05).

**Figure 6:**
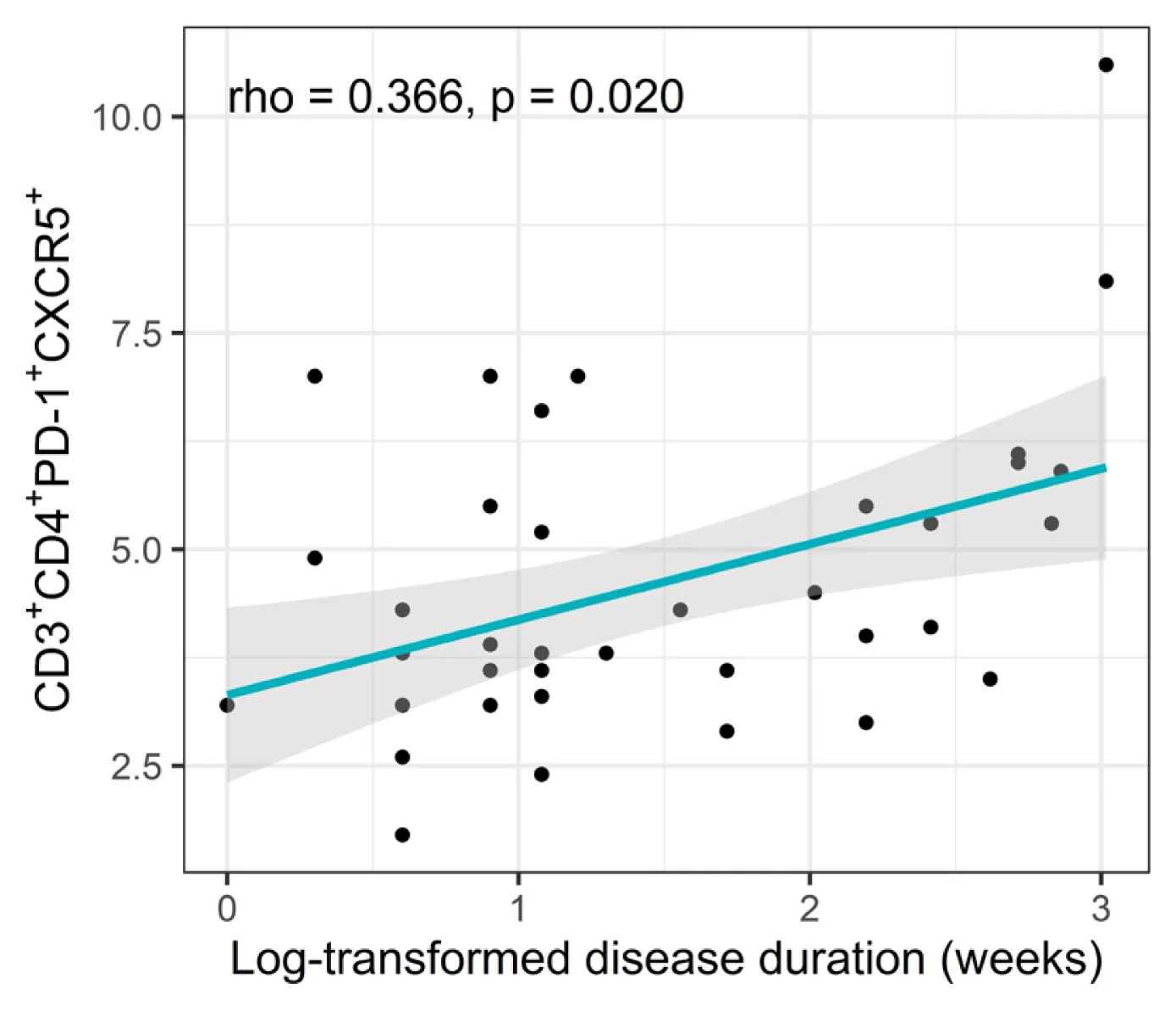
Scatter plots showing the correlation between disease duration and CD4^+^ Tfh cells. The *ρ* value represents Spearman’s rho correlation coefficient. The blue line represents the linear regression line, and the gray area represents the confidence interval.

The relationships between the clinical findings of patients with AA and T-cell subsets are presented in Table 3. CD4^+^ Tfh cells were significantly positively correlated with disease duration (Spearman’s rho = 0.366; p = 0.002; Figure 6).

**Figure 7:**
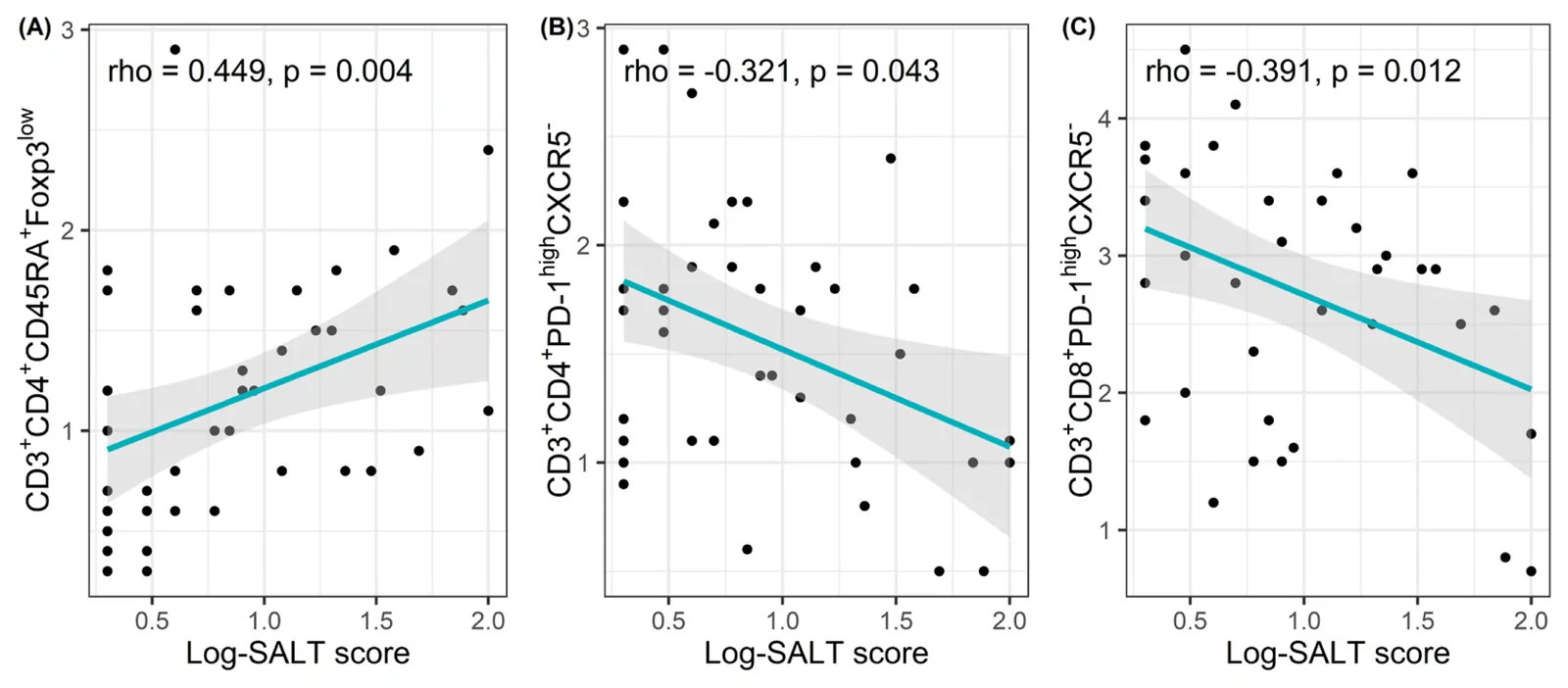
Scatter plots showing the correlation between the SALT score and (A) CD4^+^ resting Treg cells, (B) CD4^+^ Tph cells, and (C) CD8^+^ Tph cells. The ρ value represents Spearman’s rho correlation coefficient. The blue line represents the linear regression line, and the gray area represents the confidence interval.

The number of resting Treg cells increased with increasing SALT score (Spearman’s rho = 0.449; p = 0.004), whereas the number of CD4^+^ and CD8^+^ Tph cells decreased (CD4^+^: Spearman’s rho = −0.321, p = 0.043; CD8^+^: Spearman’s rho = −0.391, p = 0.012) (Figures 7A–7C). In the GLM analyses for SALT score, significant positive associations were identified for CD4^+^ Treg cells (OR: 1.467; 95% CI: 1.010–2.130), resting Treg cells (OR: 2.007; 95% CI: 1.437–2.803), CD3^+^IL-4^+^ cells (OR: 1.564; 95% CI: 1.134–2.156), and Tc2 cells (OR: 1.526; 95% CI: 1.107–2.104), whereas CD4^+^ Tph cells (OR: 0.549; 95% CI: 0.386–0.781) and CD8^+^ Tph cells (OR: 0.571; 95% CI: 0.403–0.808) showed significant inverse associations.

**Figure 8:**
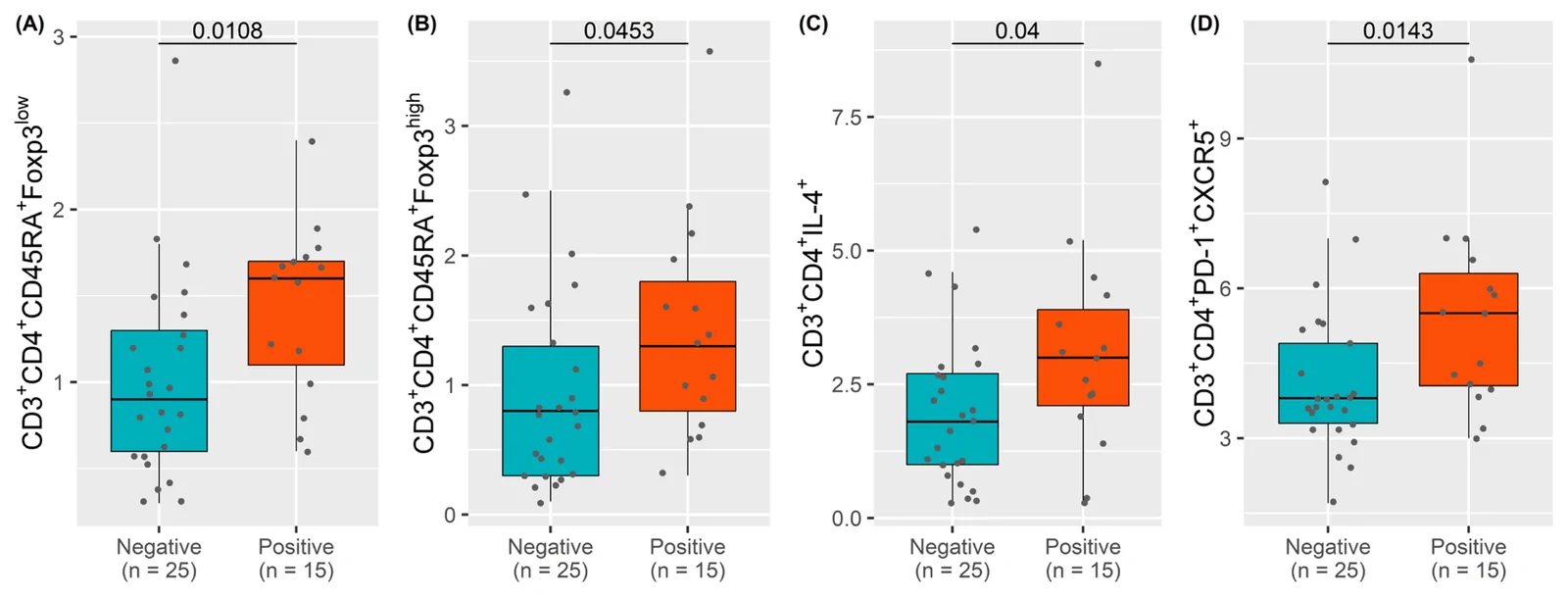
Box plots showing (A) CD4^+^ resting Treg cells, (B) CD4^+^ active Treg cells, (C) Th2 cells, and (D) CD4^+^ Tfh cells based on hair pull test results.

In univariate comparisons, patients with a positive pull test had significantly greater frequencies of CD4^+^ resting Treg cells (p = 0.011), CD4^+^ active Treg cells (p = 0.045), Th2 cells (p = 0.040), and CD4^+^ Tfh cells (p = 0.014) than those with a negative pull test. No other subpopulations showed significant differences (p > 0.05 for all) (Figures 8A–8D). After adjustment, CD4^+^ resting Treg cells and CD4^+^ Tfh cells demonstrated trend-level associations (adjusted p values of 0.067 and 0.086, respectively).

In multiple logistic regression analyses, several T-cell subsets remained independently associated with hair pull test positivity after adjustment for age, sex, disease duration, and SALT score. Notably, CD8^+^ cells (OR: 1.157; 95% CI: 1.002–1.337), resting Treg cells (OR: 5.574; 95% CI: 1.053–29.511), active Treg cells (OR: 2.827; 95% CI: 1.075–7.432), nonsuppressive Treg cells (OR: 2.847; 95% CI: 1.103–7.350), CD3^+^IFN-γ^+^ cells (OR: 1.104; 95% CI: 1.007–1.211), Th1 cells (OR: 1.136; 95% CI: 1.006–1.282), and Th2 cells (OR: 1.792; 95% CI: 1.048–3.064) demonstrated statistically significant positive associations, as indicated by confidence intervals not crossing unity.

## 4. Discussion

Despite increasing knowledge about AA, its pathogenesis and molecular mechanisms have not yet been fully elucidated. Nevertheless, AA is well recognized as an autoimmune inflammatory disease triggered by environmental factors in genetically susceptible individuals. Several studies have suggested that AA is associated with an imbalance between circulating T lymphocytes and natural killer (NK) cells. However, evidence regarding the involvement and mechanisms of T-cell subsets remains limited [9,10].

In this study, CD4^+^ and CD8^+^ T-cell subsets were comprehensively analyzed in patients with AA and healthy controls using flow cytometry. Although no significant differences were observed in the overall distributions of T-cell subsets—including total T cells; Th1, Th2, Th17, Th22, CD4^+^ and CD8^+^ Treg cells; and Tfh and Tph cells—significantly reduced frequencies of IL-22–producing total T cells, Tc17 cells, and Tc22 cells were observed in patients with AA. After adjustment, Tc17 and Tc22 remained statistically significant. Interestingly, CD4^+^ Tfh cell levels were found to be positively correlated with disease duration. Additionally, a positive association was observed between SALT scores and CD4^+^ resting Treg cells, whereas inverse associations were observed for CD4^+^ and CD8^+^ Tph cells. In the GLM analyses for SALT score, significant positive associations were identified for CD4^+^ Treg cells, resting Treg cells, CD3^+^IL-4^+^ cells, and Tc2 cells, whereas CD4^+^ Tph cells and CD8^+^ Tph cells showed significant inverse associations. Moreover, significantly elevated levels of CD4^+^ resting Treg cells, CD4^+^ activated Treg cells, Th2 cells, and CD4^+^ Tfh cells were observed in patients with a positive hair pull test. After adjustment, CD4^+^ resting Treg cells and CD4^+^ Tfh cells demonstrated trend-level associations. In multiple logistic regression analysis, CD8^+^ cells, CD4^+^ Treg subsets, CD3^+^IFN-γ^+^ cells, Th1 cells, and Th2 cells were significantly associated with positive pull test results.

CD4^+^ T cells are central to immune regulation and are involved in the activation of B cells, CD8^+^ T cells, mast cells, and macrophages following differentiation into specialized subtypes [11]. Hair follicle destruction in AA has been shown to require the coordinated activity of both CD4^+^ and CD8^+^ T cells. In AA, CD4^+^ T cells are observed to exhibit peribulbar infiltration, whereas CD8^+^ T cells are observed to target the intrabulbar region. In more severe disease, increased infiltration of both subsets into the bulge region has been reported [12]. In the present study, no significant differences were found in the mean levels of total CD4^+^ or CD8^+^ T cells between patients and controls. Consistent with these findings, no significant differences in peripheral total CD4^+^ and CD8^+^ T cells between patients with AA and healthy individuals have been reported in some studies [13,14]. Conversely, lower CD4^+^, CD8^+^, and NK cell counts in patients with AA have been reported by Younes et al. [15]. These differences may be attributable to variations in disease stage or population characteristics. Additionally, CD8^+^ cell percentages were significantly associated with a positive pull test, which is described as an indicator of active disease in the present study, and may indicate increased systemic immunological activity.

Th1 cells and IFN-γ are traditionally viewed as central to AA pathogenesis, as are other autoimmune disorders [16]. Elevated levels of serum Th1 cytokines have been documented [17].

However, more recent findings also implicate Th2, Tc2, Th17, and Th22 cells in AA pathophysiology [18–20]. In the present study, the overall frequencies of T-cell subsets were not significantly altered in patients with AA. However, multivariate analysis revealed that several immune cell subsets were independently associated with disease activity. Specifically, CD3^+^IFN-γ^+^ cells, Th1 cells, and Th2 cells were associated with positive pull test results, whereas CD^+^IL-4^+^ cells and Tc2 cells were positively associated with SALT scores. These findings suggest that both Th1-related and Th2-related immune responses contribute to disease activity in AA. The simultaneous involvement of these pathways supports the concept that AA is not driven by a single dominant immune axis but rather reflects a complex and dynamic interplay between proinflammatory and regulatory immune mechanisms.

In a previous study, total Th1 frequencies were reported to remain unchanged, whereas Th17 cells were significantly elevated in patients with AA compared to healthy controls [14]. A systemic imbalance among T helper cell subsets, characterized by increased Th1 and Th17 responses, a concomitant reduction in Treg activity, elevated levels of IL-17 and IL-22, and decreased IL-35 levels, has been shown to correlate with disease severity, supporting the role of Th17/Treg imbalance in AA progression [21].

Th22 cells and their cytokine IL-22 play essential roles in maintaining the skin barrier and are involved in inflammatory skin diseases [22]. In the present study, the levels of Tc17 and Tc22 cells were reduced in patients with AA, which may suggest the potential recruitment or sequestration of these cells within affected hair follicles. However, because only peripheral blood samples were evaluated and scalp biopsy or tissue-level immunophenotyping was not included, this interpretation should be considered speculative. Alternative explanations may include systemic immune redistribution, cytokine network alterations, or immune exhaustion mechanisms. Significant infiltration of Th1 and Th17 cells in affected hair follicles has been reported in a previous study, with Th17 cells becoming more dominant in severe disease [12]. Increased CLA^+^ Tc2 cells—indicative of skin homing—have been reported in patients with AA by Czarnowicki et al. [18]. However, studies specifically addressing Tc22 cells remain limited. These findings suggest a potential impairment in IL-22–mediated immune responses in AA.

Imbalances between regulatory and effector T-cell functions have been proposed as potential contributors to AA [10]. Animal studies have shown that Treg cells suppress AA development, whereas impaired Treg function is observed in patients with AA [23,24]. In some studies, Treg frequencies were reported to be lower in patients than in controls, whereas aTreg and rTreg levels were greater in patients with early–stage disease but decreased over time [5,25,26]. Elevated total Treg cells in peripheral blood but decreased suppressive Treg subsets have been reported by Hamed et al. [27]. Immunohistochemical analyses have revealed lower Treg densities in lesional skin than in nonlesional or healthy skin, with suppressive Treg cells preferentially localizing to nonlesional follicles. In the present study, no significant differences were observed in CD4^+^ Treg cells or their subgroups or in CD8^+^ Treg cells between patients and healthy controls. However, CD4^+^ Treg subsets were significantly elevated in patients with a positive pull test compared with those with a negative test. Moreover, CD4^+^ Treg and resting Treg subset levels increased in parallel with higher SALT scores, reflecting greater disease severity. These findings may reflect an immunoregulatory response attempting to counteract heightened immune activity during active disease phases. Interestingly, increased Treg activity has been observed in certain AA variants, such as acute diffuse alopecia, which may contribute to a more favorable prognosis, suggesting that Treg function plays a critical role in disease modulation rather than simple deficiency alone [28].

The role of humoral immunity in AA is less well defined. A Th2–dominant profile has been identified in some patients with AA in recent serum and skin studies. The frequent coexistence of AA and atopic dermatitis suggests shared Th2–driven mechanisms [29]. A positive correlation between Th2 cell levels and disease severity has been reported in some studies [18,30]. However, Th1 cells also inhibit Th2 responses, complicating the interpretation of the role of Th2 cells in AA [29].

T follicular (Tfh) cells are a CD4^+^ T-cell subset that supports B-cell maturation and antibody production. Upon activation in lymphoid organs, a subset of these cells enters the peripheral circulation as Tph cells. These cell subsets have been implicated in chronic inflammation in systemic autoimmune diseases [31–33]. In the present study, CD4^+^ Tfh cell levels increased with disease duration, indicating their possible involvement in disease chronicity or persistence. CD4^+^ and CD8^+^ Tph cells decreased with increasing SALT scores. Collectively, these findings underscore the involvement of CD4^+^ Tfh and Tph cells in the immune dysregulation observed during both the active and chronic phases of AA.

This study has several limitations. The single-center design, cross-sectional approach, and relatively small sample size limit generalizability, preclude causal inference, and may reduce the power of subgroup analyses. Furthermore, the absence of pediatric or severe AU cases and the lack of cytokine functional assays restrict the interpretation of the findings. Because most patients had patch-type alopecia areata and only a small number had other clinical phenotypes, subgroup analyses according to disease phenotype were not feasible. This heterogeneity may influence immune profiles and should be addressed in larger studies. Future multicenter studies integrating both tissue and peripheral immunophenotyping with longitudinal follow-up are needed to better clarify these mechanisms.

In conclusion, a comprehensive characterization of circulating T-cell subsets in patients with AA is provided, highlighting significant immune dysregulation across both CD4^+^ and CD8^+^ populations. A decrease in peripheral Tc17 and Tc22 cells was observed. Additionally, the percentages of CD8^+^ cells, CD3^+^IFN-γ^+^ cells, Th1 cells, and Th2 cells were significantly associated with a positive pull test, which may indicate increased systemic immunological activity. CD3^+^IL-4^+^ cells and Tc2 cells showed significant positive associations with SALT scores. These findings indicate that selected cytokine-producing T-cell subsets may serve as independent immunological correlates of disease activity, highlighting the multifaceted nature of immune dysregulation in AA.

Elevated CD4^+^ Tfh cells and their associations with disease duration suggest a role in immune dysregulation during chronic phases. An increase in CD4^+^ Treg subsets in patients with active disease and higher SALT scores suggests a potential regulatory feedback response. A decrease in CD4^+^ and CD8^+^ Tph cells with increasing disease severity may reflect the modulation of antibody responses.

Collectively, these findings highlight the complex and dynamic nature of T-cell–mediated immune regulation in AA. T-cell subset profiling may provide preliminary insights into disease activity and immune dysregulation; however, longitudinal studies are required before clinical application. In the future, larger cohorts including patients with more severe disease and diverse AA phenotypes, including pediatric patients, should be investigated.

## Supplementary materials

**Supplementary Table 1:** Evaluation of T cell subsets based on cell surface receptors or cytokine expression.

T cell subsets	Monoclonal antibodies
Total T cell	CD3 ^+^
IFN- *γ* ^+^ total T cell	CD3 ^+^IFN- *γ* ^+^
IL-4 ^+^ total T cell	CD3 ^+^IL-4 ^+^
IL-17 ^+^ total T cell	CD3 ^+^IL-17A ^+^
IL-22 ^+^ total T cell	CD3 ^+^IL-22 ^+^
T helper cell	CD3 ^+^ CD4 ^+^
T helper 1	CD3 ^+^ CD4 ^+^ IFN-γ ^+^
T helper 2	CD3 ^+^ CD4 ^+^ IL-4 ^+^
T helper 17	CD3 ^+^ CD4 ^+^ IL-17A ^+^
T helper 22	CD3 ^+^ CD4 ^+^ IL-22 ^+^
CD4 ^+^ T regulatory cell	CD3 ^+^ CD4 ^+^ CD25 ^+^ Foxp3 ^+^
Resting T regulatory cell	CD3 ^+^ CD4 ^+^ CD45RA ^+^ Foxp3 ^low^
Active T regulatory cell	CD3 ^+^ CD4 ^+^ CD45RA ^−^ Foxp3 ^high^
Nonsuppressive T regulatory cell	CD3 ^+^ CD4 ^+^ CD45RA ^−^Foxp3 ^low^
CD4 ^+^ follicular T cell (Tfh)	CD3 ^+^ CD4 ^+^ PD1 ^+^ CXCR5 ^+^
CD4 ^+^ peripheral T cell (Tph)	CD3 ^+^ CD4 ^+^ PD1 ^high^ CXCR5 ^−^
Cytotoxic T cell	CD3 ^+^ CD8 ^+^
Cytotoxic T1	CD3 ^+^ CD8 ^+^ IFN-γ ^+^
Cytotoxic T2	CD3 ^+^ CD8 ^+^ IL-4 ^+^
Cytotoxic T17	CD3 ^+^ CD8 ^+^ IL-17A ^+^
Cytotoxic T22	CD3 ^+^ CD8 ^+^ IL-22 ^+^
CD8 ^+^ regulatory T cell	CD3 ^+^ CD8 ^+^ CD25 ^+^ Foxp3 ^+^
CD8 ^+^ follicular T cell (Tfh)	CD3 ^+^ CD8 ^+^ PD1 ^+^ CXCR5 ^+^
CD8 ^+^ peripheral T cell (Tph)	CD3 ^+^ CD8 ^+^ PD1 ^high^ CXCR5 ^−^

**Supplementary Table 2:** Antibody clones, their conjugated fluorescences.

Monoclonal Antibodies	Dyes	Clones	Manufacturers
CD3	PerCP-Cy5	OKT3	BD Bioscience, CA, USA
CD4	APC	RPA-T4	BD Bioscience, CA, USA
CD8	Alexa Fluor 700	RPA-T8	BD Bioscience, CA, USA
Foxp3	FITC	206D	BD Bioscience, CA, USA
CD25	PE	BC96	BD Bioscience, CA, USA
CD45RA	APC-Cy7	HI100	BD Bioscience, CA, USA
CD3/CD4/CD8	PE-Cy5/PE/FITC	UCHT1/RPA-T4/RPA-T8	BioLegend, CA, USA
CXCR5 (CD185)	FITC	J252D4	BioLegend, CA, USA
PD-1 (CD279)	PE-Cy7	EH12.2H7	BioLegend, CA, USA
IL-4	PE-Cy7	MP4-25D2	BioLegend, CA, USA
IL-17A	APC	BL168	BioLegend, CA, USA
IL-22	APC	2G12A41	BioLegend, CA, USA
IFN-γ	APC	4SB3	BioLegend, CA, USA

Supplementary Figure 1 Flow cytometric analysis of CD4^+^ (Th1, Th2, Th17, Th22) and CD8^+^ (Tc1, Tc2, Tc17, Tc22) T-cell subsets.

Supplementary Figure 2 Flow cytometric analysis of CD4^+^ and CD8^+^ follicular and peripheral T-cell subsets.

Supplementary Figure 3 Flow cytometry gating strategy for regulatory T cells. **A** CD25^+^ Foxp3^+^ cells were gated from CD4^+^ and CD8^+^ lymphocytes. **B** I: CD3^+^ CD4^+^ CD45RA^+^ Foxp3^low^ (resting Tregs), II: CD3^+^ CD4^+^ CD45RA^−^ Foxp3^high^ (active Tregs), III: CD3^+^ CD4^+^ CD45RA^−^ Foxp3^low^ (nonsuppressive Treg).

## Figures and Tables

**Table 1:** Demographic, clinical, and laboratory characteristics of study participants.

Characteristics	Controls (n = 40)	Patients (n = 40)	p-values
**Demographic characteristics**			
Age (years), mean ± SD (min–max)	29.85 ± 11.06 (17–54)	30.75 ± 11.97 (16–55)	0.728^1^
Sex (female/male), n (%)	16 (40) / 24 (60)	13 (32.5) / 27 (67.5)	0.642^2^
**Clinical characteristics**			
Alopecia areata (patch), n (%)		36 (90)	
Alopecia totalis, n (%)		1 (2.5)	
Alopecia universalis, n (%)		1 (2.5)	
Ophiasis, n (%)		2 (5)	
Disease duration (weeks), median (min–max)		12 (1–1040)	
SALT score, median (min–max)		6.5 (2–100)	
SALT score staging (S1/S2/S3/S4a/S5), n (%)		32 (80) / 4 (10) / 1 (2.5) / 1 (2.5) / 2 (5)	
Pull test (positive), n (%)		15 (37.5)	
Nail involvement, n (%)		0	
**Hematological parameters**			
WBCs, mean ± SD		8073.25 ± 1812.58	
Neutrophils, mean ± SD		4866.25 ± 1418.82	
Neutrophils (%), mean ± SD		59.53 ± 7.87	
Monocytes, mean ± SD		59.92 ± 19.54	
Monocytes (%), mean ± SD		7.62 ± 2.02	
Lymphocytes, mean ± SD		2413.25 ± 693.30	
Lymphocytes (%), mean ± SD		30.27 ± 7.08	

1 Independent samples t-test.

2 Chi-square test with Yates continuity correction.

**Table 2:** Comparison of T-cell subsets between study groups.

T cell subsets (%)	Controls (n = 40)	Patients (n = 40)	p-values	Adjusted p-values (FDR)
CD3^+^	71.83 ± 6.74	69.29 ± 6.37	0.087^1^	0.184
CD3^+^CD4^+^	39.85 (36.77–45.42)	40.15 (35.80–44.02)	0.927^2^	0.927
CD3^+^CD8^+^	30.95 ± 7.65	28.30 ± 6.54	0.099^1^	0.184
CD3^+^CD4^+^CD25^+^Foxp3^+^	3.40 (3.08–4.55)	3.15 (2.38–4.45)	0.242^2^	0.387
CD3^+^CD8^+^CD25^+^Foxp3^+^	0.10 (0.10–0.23)	0.10 (0.10–0.20)	0.194^2^	0.356
CD3^+^CD4^+^CD45RA^+^Foxp3^low^	1.23 ± 0.50	1.17 ± 0.59	0.640^1^	0.799
CD3^+^CD4^+^CD45RA^−^Foxp3^high^	0.80 (0.50–1.13)	0.85 (0.47–1.60)	0.429^2^	0.613
CD3^+^CD4^+^CD45RA^−^Foxp3^low^	2.80 (2.30–3.58)	3.45 (2.90–3.90)	0.063^2^	0.168
CD3^+^IFN-*γ*^+^	15.71 ± 8.29	16.06 ± 9.35	0.860^1^	0.912
CD3^+^CD4^+^IFN-*γ*^+^	11.90 (7.32–14.75)	11.90 (7.30–15.20)	0.844^2^	0.912
CD3^+^CD8^+^IFN-*γ*^+^	23.80 (13.62–33.88)	19.30 (11.78–26.95)	0.402^2^	0.603
CD3^+^IL-4^+^	1.55 (0.88–2.92)	1.50 (0.78–2.30)	0.586^2^	0.761
CD3^+^CD4^+^ IL-4^+^	2.05 (1.00–3.42)	2.25 (1.08–3.13)	0.923^2^	0.927
CD3^+^CD8^+^ IL-4^+^	1.45 (1.00–2.80)	1.20 (0.60–2.10)	0.248^2^	0.387
CD3^+^IL-17A^+^	0.70 (0.38–1.00)	0.50 (0.20–0.95)	0.149^2^	0.320
CD3^+^CD4^+^ IL-17A^+^	0.85 (0.50–1.33)	0.75 (0.30–1.33)	0.412^2^	0.603
**CD3** **^+^** **CD8** **^+^** ** IL-17A** **^+^**	0.50 (0.30–0.80)	0.20 (0.10–0.50)	**0.003** 2	**0.003**
**CD3** **^+^** **IL-22** **^+^**	0.60 (0.40–1.33)	0.40 (0.20–1.10)	**0.043** 2	0.148
CD3^+^CD4^+^ IL-22^+^	0.90 (0.57–1.65)	0.75 (0.30–1.40)	0.177^2^	0.356
**CD3** **^+^** **CD8** **^+^** ** IL-22** **^+^**	0.60 (0.30–0.90)	0.20 (0.20–0.40)	**<0.001** 2	**0.012**
CD3^+^CD4^+^PD-1^+^CXCR5^+^	3.90 (3.40–4.32)	3.95 (3.58–5.50)	0.240^2^	0.387
CD3^+^CD8^+^PD-1^+^CXCR5^+^	1 (0.70–1.33)	0.85 (0.57–1.22)	0.318^2^	0.474
CD3^+^CD4^+^PD-1^high^CXCR5^−^	1.35 ± 0.47	1.56 ± 0.61	0.085^3^	0.184
CD3^+^CD8^+^PD-1^high^CXCR5^−^	2.54 ± 0.82	2.78 ± 0.95	0.234^1^	0.387

Data are presented as mean ± standard deviation or median (IQR: 1st–3rd quartile).

Adjusted p-values were calculated using the Benjamini–Hochberg false discovery rate (FDR) method. Statistically significant values are shown in bold.

1 Independent samples t-test.

2 Mann–Whitney U test.

3 Welch’s t-test.

**Table 3:** Associations between clinical findings and T-cell subsets.

T cell subsets (%)	Disease duration^1^	SALT scores^1^	Generalized linear model	Pull test		p-value	Adjusted p-values (FDR)	Multiple logistic regression analyses

				Negative (n = 25)	Positive (n = 15)			

CD3^+^	0.030	−0.063	1.046 (0.633–1.728)	69.03 ± 5.35	69.73 ± 7.98	0.743^2^	0.912	0.945 (0.828–1.079)
CD3^+^CD4^+^	−0.054	0.163	1.451 (0.914–2.302)	40.5 (38.6–44.7)	38.4 (34.05–42.45)	0.219^3^	0.384	0.917 (0.823–1.022)
CD3^+^CD8^+^	−0.040	−0.239	0.772 (0.479–1.244)	25.1 (24.1–29.1)	31.5 (25.8–37.05)	0.088^3^	0.177	**1.157 (1.002–1.337)** *
CD3^+^CD4^+^CD25^+^Foxp3^+^	−0.141	0.036	**1.467 (1.010–2.130)** *	3.29 ± 1.62	3.83 ± 1.69	0.322^2^	0.497	1.590 (0.948–2.667)
CD3^+^CD8^+^CD25^+^Foxp3^+^	−0.014	−0.046	0.994 (0.618–1.600)	0.10 (0.10–0.20)	0.10 (0.10–0.20)	0.341^3^	0.506	22.605 (0.060–8467.225)
Resting Treg CD3^+^CD4^+^CD45RA^+^ Foxp3^low^	0.120	**0.449** *	**2.007 (1.437–2.803)** *	0.90 (0.60–1.30)	1.60 (1.10–1.70)	**0.011** 3	0.067	**5.574 (1.053–29.511)** *
Active Treg CD3^+^CD4^+^CD45RA^−^Foxp3^high^	0.061	0.212	1.344 (0.855–2.112)	0.80 (0.30–1.30)	1.30 (0.80–1.80)	0.**045**^3^	0.181	**2.827 (1.075–7.432)** *
Nonsuppressive Treg CD3^+^ CD4^+^CD45RA^−^Foxp3^low^	−0.247	0.059	1.352 (0.854–2.139)	3.24 ± 1.03	3.71 ± 1.14	0.187^2^	0.384	**2.847 (1.103–7.350)** *
CD3^+^IFN-*γ*^+^	−0.066	0.232	1.361 (0.880–2.106)	14.10 ± 7.63	19.34 ± 11.20	0.086^2^	0.177	**1.104 (1.007–1.211)** *
CD3^+^CD4^+^IFN-*γ*^+^	−0.073	0.209	1.380 (0.899–2.117)	10.29 ± 5.43	14.48 ± 8.39	0.062^2^	0.177	**1.136 (1.006–1.282)** *
CD3^+^CD8^+^IFN-*γ*^+^	−0.116	0.204	1.369 (0.896–2.092)	16.2 (10–24.6)	21.2 (16.35–38.25)	0.332^3^	0.497	1.046 (0.997–1.097)
CD3^+^IL-4^+^	−0.012	0.243	**1.564 (1.134–2.156)** *	1.2 (0.6–2.2)	1.8 (1.25–2.8)	0.235^3^	0.397	1.557 (0.934–2.594)
CD3^+^CD4^+^ IL-4^+^	−0.075	0.099	1.372 (0.955–1.972)	1.95 ± 1.38	3.10 ± 2.04	**0.040** 2	0.181	**1.792 (1.048–3.064)** *
CD3^+^CD8^+^ IL-4^+^	−0.306	−0.019	**1.526 (1.107–2.104)** *	0.9 (0.6–2.1)	1.4 (0.65–2.10)	0.595^3^	0.794	1.388 (0.960–2.006)
CD3^+^IL-17A^+^	−0.020	0.093	0.914 (0.763–1.097)	0.4 (0.2–0.7)	0.7 (0.45–1.20)	0.057^3^	0.177	2.434 (0.667–8.877)
CD3^+^ CD4^+^ IL-17A^+^	−0.034	0.072	0.788 (0.482–1.289)	0.6 (0.3–1.1)	1 (0.6–1.45)	0.085^3^	0.177	2.036 (0.670–6.186)
CD3^+^CD8^+^ IL-17A^+^	0.258	0.056	0.813 (0.496–1.333)	0.2 (0.1–0.4)	0.3 (0.15–0.85)	0.143^3^	0.286	7.636 (0.984–59.255)
CD3^+^IL-22^+^	−0.126	0.137	0.750 (0.480–1.171)	0.3 (0.1–0.8)	0.7 (0.35–1.60)	0.051^3^	0.177	2.084 (0.835–5.198)
CD3^+^CD4^+^ IL-22^+^	−0.158	0.088	0.861 (0.544–1.362)	0.5 (0.2–1.2)	1.3 (0.7–1.45)	0.107^3^	0.206	1.842 (0.830–4.088)
CD3^+^CD8^+^ IL-22^+^	−0.005	−0.057	1.055 (0.647–1.722)	0.2 (0.2–0.4)	0.2 (0.2–0.6)	0.690^3^	0.891	0.489 (0.053–4.525)
CD3^+^CD4^+^PD-1^+^CXCR5^+^	**0.366** *	0.306	1.177 (0.657–2.108)	3.8 (3.3–4.9)	5.5 (4.05–6.30)	**0.014** 3	0.086	1.831 (0.980–3.423)
CD3^+^CD8^+^PD-1^+^CXCR5^+^	−0.166	0.134	1.343 (0.839–2.152)	0.9 (0.6–1.2)	0.8 (0.5–1.3)	0.999^3^	0.999	1.554 (0.466–5.176)
CD3^+^CD4^+^PD-1^high^CXCR5^−^	−0.289	**−0.321** *	**0.549 (0.386–0.781)** *	1.62 ± 0.65	1.47 ± 0.54	0.481^2^	0.694	0.823 (0.200–3.390)
CD3^+^CD8^+^PD-1^high^CXCR5^−^	−0.120	**−0.391** *	**0.571 (0.403–0.808)** *	2.80 ± 1.02	2.75 ± 0.84	0.876^2^	0.962	1.369 (0.513–3.654)

1 Spearman’s rho correlation coefficient;

2 Independent samples t-test;

3 Mann–Whitney U test.

* Correlation analysis results indicate statistically significant values (p < 0.05).

Statistically significant values are shown in bold.

## Data Availability

The data that support the findings of this study are available from the corresponding author upon reasonable request. The data are not publicly available due to privacy and ethical restrictions.
